# Is There a Functional Role of Mitochondrial Dysfunction in the Pathogenesis of ARPKD?

**DOI:** 10.3389/fmed.2021.739534

**Published:** 2021-10-05

**Authors:** Max Christoph Liebau

**Affiliations:** Department of Pediatrics, Center for Molecular Medicine, and Center for Rare Diseases, University Hospital Cologne and Medical Faculty, University of Cologne, Cologne, Germany

**Keywords:** PKD, fibrocystin, *PKHD1*, *PKD1*, *PKD2*, polycystin

## Introduction

Polycystic kidney diseases (PKD) form a group of severe genetic kidney diseases that are characterized by fibrocystic changes of the kidneys and the liver. The main forms of PKD are autosomal dominant and autosomal recessive polycystic kidney disease (ADPKD, ARPKD). In general, cystic kidney diseases are the most common genetic cause of chronic kidney failure in both children and adults (1).

ARPKD is a disorder that typically is diagnosed very early in life or even prenatally. Typically, the kidneys show bilateral massive enlargement although they keep their reniform shape. Kidney disease results in a need for kidney replacement therapy (KRT) in around 50% of patients in the first two decades of life (2, 3). Liver involvement is obligatory in ARPKD, typically presenting with congenital hepatic fibrosis and portal hypertension or with dilated bile ducts. Both are considered to be a consequence of a defect during development of the bile ducts (“ductal plate malformation”) (4). Adult patients with ARPKD have been described with variable disease manifestations. Hepatic disease may be the leading clinical issue in adults with ARPKD (5).

In ADPKD renal cystogenesis starts early in life or even before birth but does not present with obvious clinical symptoms until adulthood in most patients (1, 6, 7). Liver involvement is typically characterized by liver cysts. Additional extrarenal symptoms in ADPKD include for example intracranial aneurysms, cardiac valve anomalies like mitral prolapse or abdominal wall diverticula in a subset of patients (1, 7). Extrarenal manifestations may be very helpful for clinical differential diagnosis and to distinguish ADPKD from ARPKD.

ADPKD is mainly caused by variants in the genes *PKD1* and *PKD2* with the large majority of patients carrying *PKD1* variants (1). Variants in other genes e.g., with atypical forms of ADPKD have been identified (7). Variants in *PKHD1* are the main cause of ARPKD with phenotypical overlay e.g., of kidney disease in patients with other underlying genetic changes (1, 8), including biallelic hypomorphic *PKD1* variants.

Genotype-phenotype correlations in ADPKD show more rapid progression of kidney disease in patients with *PKD1* variants and especially in those patients carrying variants resulting in protein truncation (7, 9). For ARPKD it was shown by various groups that biallelic truncating variants are associated with severe phenotypes. A recent study extended the findings and revealed that for missense variants the affected region in *PKHD1* also seems to be important (10). In a study on 304 children with the clinical diagnosis of ARPKD and detected *PKHD1* variants it was found that patients with either two missense variants affecting the amino acids 709–1837 or a null variant and a missense variant in this region less frequently showed progression to chronic kidney failure when being compared during their follow-up when compared to patients with variants affecting other regions of *PKHD1*. On the other hand patients with variants affecting the amino acids 2625–4074 showed less favorable hepatic outcome (10). The underlying molecular mechanisms of these associations remain to be explored.

## Cellular Functions of ADPKD Proteins

Even though many questions remain, much has been learned about the functions of polycystin-1 (PC1) and polycystin-2 (PC2), the proteins encoded by *PKD1* and *PKD2*, respectively. Very briefly summarized, PC1 is a very large transmembrane receptor-like protein with 11 transmembrane domains and a large extracellular part. PC2 is non-selective cation channel and belongs to the family of transient receptor potential ion channels (TRPs). Both PC proteins have been found in a joint protein complex but may also have independent cellular functions (11). Recent work on high-resolution cryo-electron microscopy has found that the transmembrane domains of PC1 and PC2 can assemble in a structure consisting of three PC2 molecules and one PC1 molecule. Still, many questions on how the proteins interact remain unsolved (12).

PC1 and PC2 have been shown to be involved in the regulation of multiple signaling networks that obviously are closely interconnected and cannot be seen as isolated events. There is e.g., dysregulation of intracellular cAMP signaling in ADPKD which itself is controlled by the vasopressin V2 receptor. Insights into a dysregulation of this cascade have led to the establishment of the first targeted treatment in ADPKD for adult patients with the use of the V2 receptor antagonist Tolvaptan (1, 7, 13). Other dysregulated signaling cascades include tyrosine receptor kinase-PKA-SRC signaling, Myc- and STAT-signaling or macrophage-activation (11).

Recently, evidence for a role of PC1 in the regulation of cellular metabolic processes has been accumulating. Details of the work have recently been reviewed and are beyond the scope of this manuscript, but it has been shown that PC1 is involved in the regulation of multiple signaling cascades controlling cellular metabolic processes (14, 15). This includes regulation of mTOR signaling, amino acid and glutamine metabolism as well as changes in fatty acid oxidation (14, 15). The ADPKD proteins PC1 and PC2 seem to be crucial regulators of cellular metabolism including effect on the cellular sensor AMPK, the transcription factor PPARalpha and the regulator of mitochondrial biosynthesis PPARγ Coaktivator 1α (PGC1α) or the tumor suppressor kinase LKB1. A landmark study that served as a starting point for intensive follow-up studies identified a shift of glycolysis and oxidative phosphorylation and a Warburg-effect like effect in PKD cells (16). An increase of glycolysis with altered fatty acid oxidation and mitochondrial function as well as an increase of reactive oxygen species have independently been identified in various cellular and preclinical models as well as in samples from patients (15–22). In addition to these metabolic alterations, changes in mitochondrial structure from PKD cells were identified and fragments of the cytoplasmic PC1 tail or the PC1–PC2 complex have been shown to localize to mitochondria and mitochondria-associated membranes (18, 23).

Importantly, these findings bear therapeutic potential. In a preclinical setting inhibition of cellular glycolysis by the non-metabolizable 2-Deoxyglucose (2-DG) or caloric restriction results in reduced cyst growth in both orthologous and non-orthologous mouse models of ADPKD (15–17, 24). Furthermore, for an activation of PPARα (Fenofibrate), PPARγ (Glitazone) and AMPK (Metformin) positive effects have been described (15). A phase 2 trial recently found that metformin was safe and tolerable in adult patients in early ADPKD stages resulting in a slight reduction of eGFR loss that however did not yet reach significance in this trial of 97 patients (25). A larger trial will be required. Follow-up work on caloric restriction studies revealed that also time-restricted feeding induced positive effects (26). A ketogenic diet but also oral administration of the ketone ß-hydroxybutyrate showed very promising effects on the kidney phenotype in various preclinical PKD models (26). Translation of these preclinical findings into clinical trials is ongoing.

## Overlap Between ADPKD and ARPKD

Various lines of evidence point to an overlap of pathogenic factors between ARPKD and ADPKD (Table 1). As previously pointed out patients with (biallelic) *PKD1* variants may mimic the ARPKD phenotype, even though ARPKD is typically caused by variants in *PKHD1*. Furthermore, patients with variants in multiple PKD genes and preclinical rodent models with digenic genetic modifications show more severe phenotypes (27–29). Importantly, the PCK rat, a preclinical model that phenotypically mimics ADPKD, genetically is orthologous to ARPKD. Overlapping dysregulation of signaling cascades has been described, e.g., with STAT3 activation in cyst-lining epithelial cells in both ARPKD and ADPKD (47–49). While the main gene affected in ARPKD has been known for two decades, the function of the *PKHD1*-encoded protein fibrocystin (FC) remains incompletely understood. FC has been found in joint protein complexes with the ADPKD proteins PC1 and PC2 in extracellular vesicles (50, 51). A physical interaction has also been proposed for FC and PC2 (52) but the data has not been independently confirmed. Overall, ARPKD and ADPKD may not be seen as completely separate diseases but rather as two different ends of a spectrum of disease.

**Table 1:** Selected evidence for overlapping dysregulated signaling cascades and metabolic changes in ARPKD and ADPKD.

	***In vitro* evidence or preclinical *in vivo* evidence**	**Evidence from patients or patient samples**
Genetic interaction of ARPKD and ADPKD genes	(27, 28)	(29–31) (incl. phenocopies)
Overlapping signaling cascades incl. vasopressin-cAMP- cascade or EGFR-SRC- STAT3 signaling	Recently reviewed in (1, 32)	
**Metabolic changes in PKD**	**ARPKD**	**ADPKD**
Evidence for mTOR activation	Evidence from: - cell culture experiments - an orthologous rat model (*PCK* rat, phenotypically resembling ADPKD) - nephrectomy samples from patients (33–35)	Evidence from: - cell culture experiments - orthologous and non-orthologous rodent models - nephrectomy samples from patients (36, 37)
Evidence for enhanced glycolysis	Evidence from cell culture, genetically modified HEK293 cells (38)	Evidence from: - cell culture experiments - orthologous mouse models - nephrectomy samples from patients (16, 39)
Evidence for metabolic reprogramming of amino acid metabolism incl. glutamine dependency	Evidence from: - the CPK mouse model, a non-orthologous model of ARPKD - nephrectomy samples from patients (40)	Evidence from: - cell culture experiments - orthologous and non-orthologous rodent models - nephrectomy samples from patients (19, 41, 42)
Evidence for dysregulated fatty acid oxidation	Evidence from: - an orthologous rat model (*PCK* rat, phenotypically resembling ADPKD) - patients with an ARPKD phenotype with underlying variants in relevant genes of fatty acid oxidation (43–45)	Evidence from: - cell culture experiments - orthologous and non-orthologous rodent models - nephrectomy samples from patients (19, 20, 46)

## Fibrocystin, ARPKD and Cellular Metabolism

So, what is the evidence suggesting that there could also be a role for mitochondrial dysfunction in the pathogenesis of ARPKD? Data is much more limited for ARPKD than for ADPKD, but first hints of cellular metabolic dysregulation in ARPKD are emerging.

Importantly, an activation of mTOR in cyst-lining renal epithelial cells was described for ARPKD (33). Full length FC was shown to control mTOR activation *in vitro* with the cytoplasmic tail regulating the function of full-length protein (34). Treatment with NVP-BEZ235, an inhibitor of PI3-kinase and mTOR, had a positive effect on cystic dilatation of the intrahepatic bile ducts in the PCK rat (35).

A positive effect of the PPARγ-agonist pioglitazone on the renal and hepatic phenotype was shown in the PCK rat (43). Gene expression analyses after treatment with pioglitazone pointed to an important modification of fatty acid oxidation by this treatment in the PCK rat (44). Defects in lipid metabolism and fatty acid metabolism were described as modifiers in the same rat model that were associated with a more severe renal phenotype (53). Interestingly, defects of fatty acid oxidation in humans can result in a renal phenotype that resembles ARPKD (4, 45) and need to be considered as clinical differential diagnoses (4). In this context another interesting differential diagnosis of ARPKD are variants in the promoter region of *PMM2* encoding Phosphomannomutase 2 that can result in an ARPKD-like kidney phenotype combined with hyperinsulinemic hypoglycemia (54).

A recent publication looked at the effects of truncating *PKHD1* variants in HEK293 cells on metabolic aspects. Variants were induced by Crispr/Cas9 and were associated with an acidification of the cell medium, an activation of the Krebs cycle and mild structural changes in mitochondria (38). Even though HEK293 cells may not be fully recapitulating the specifics of human renal tubular cells this finding is of interest.

A challenge in ARPKD research in this context is the fact that orthologous mouse models do not fully recapitulate the human renal phenotypes although various models have shown hepatic effects that are closer to human disease. Non-orthologous models have given important insights (55). The recent description of digenic *Pkd1*-*Pkhd1* mouse that mimic the kidney phenotype of ARPKD was therefore of major interest (28). The experience from previous studies highlight the importance of studying mouse models for ADPKD that show the slowly progressive kidney phenotype as some of the *Pkd1* knockout models show rapid progression (56). Crossing the slowly progressive ADPKD *Pkd1*^RC/RC^ model (representing the hypomorphic human missense variant p.Arg3277Cys) variant with a *Pkhd1*^−/−^ model (only developing mild kidney disease) resulted in ARPKD-like kidneys in a mouse model. Thus, these models may be helpful to study ARPKD kidney disease in preclinical models and may turn out to be a major step ahead despite being genetically partially different from human disease (28). As an ideal orthologous model is not available such data needs to be complemented e.g., by patient samples. It was interesting to note that a proteomics approach comparing human ARPKD kidneys and control kidneys identified upregulation of many proteins localizing to mitochondria (57) and that an increase of the oxygen consumption rate was noted in HEK293 cells with truncating *PKHD1* variants whereas a reduction of mitochondrial mass has been reported in cyst-lining cells from a murine *Pkd1* model. Thus, it seems possible that there are specific *PKHD1* effects on mitochondria that may partly be different from *PKD1* or *PKD2* effects. More overlapping data from preclinical models and patient samples as well as functional work on the *PKHD1*-encoded protein fibrocystin will be required. It is important to note that the examined kidneys in the proteomics study were obtained from young patients obviously suffering from severe disease requiring nephrectomy. Such patients may show rapid and sometimes enormous growth of the kidneys. Obviously, patient samples such as nephrectomy specimens will in many cases give insights into a terminal situation of the kidneys with pronounced chronic kidney disease of the patients and differentiating cause and consequence may be a challenge. A link to clinical courses will thus also be needed. Current national and international data and sample collections (e.g., Rare Renal in the UK, the hepatorenal fibrocystic core center in the US, ARegPKD in Europe) may become important resources for such approaches.

Different from ADPKD, rapid kidney growth in ARPKD typically occurs before birth with some patients showing rapid renal growth very early in life. Indeed, many ARPKD patients are nowadays identified antenatally. A recent clinical publication identified prenatal risk markers for early dialysis dependency in ARPKD (58). These markers included the antenatal detection of enlarged kidneys and have served as the basis for a first phase 3 trials in ARPKD that are currently being initiated (NCT04782258, NCT04786574). *In utero*, kidneys are exposed to a hypoxic environment as oxygen pressures within the fetal blood are generally lower with the kidneys being located in a lethal corner at the very end of oxygen delivery. It will be interesting to study the changes of FC function under hypoxic conditions even more as e.g., the oxygen-regulated transcription factor HIF1a is known to enhance cystogenesis in an ADPKD model (59). Strikingly, HIF1 activates glycolysis and mitochondrial oxygen consumption (60). Again, there is a link to ADPKD: local hypoxia is a presumed factor contributing to kidney disease progression in ADPKD.

## Outlook and Summary

Over the past years, metabolic changes in PKD have become the focus of a highly active field of research. Most of the work has been done on ADPKD but there is first evidence that metabolic changes may also be important in ARPKD disease progression. Deciphering primary changes from secondary changes due to chronic kidney disease may become particularly challenging in ARPKD due to the early development of severe kidney disease in ARPKD. Linking functional cellular work and novel preclinical models to patient genetics, patient disease courses and biosamples seems particularly relevant to obtain a balanced view of causes and consequences in ARPKD disease progression and cellular metabolic changes. Lessons learned from ADPKD may guide the steps to take to achieve progress in our understanding of this severe disease with the potential to open paths for first targeted therapeutic approaches.

## Author Contributions

The author confirms being the sole contributor of this work and has approved it for publication.

## Funding

ML is a member of the European Reference Network on Rare Kidney Diseases (ERKNet)—Project ID No. 739532 who was supported by grants of the German Society for Pediatric Nephrology, the European Society for Paediatric Nephrology (ESPN2014.2), and the Marga and Walter Boll-Foundation, and furthermore supported by the German Federal Ministry of Research and Education (BMBF grant 01GM1903B, NEOCYST consortium) and the German Research Council (DFG Li 23975/1).

## Conflict of Interest

ML serves on an advisory board of Otsuka Pharmaceuticals as a representative of the University Hospital of Cologne.

## Publisher's Note

All claims expressed in this article are solely those of the authors and do not necessarily represent those of their affiliated organizations, or those of the publisher, the editors and the reviewers. Any product that may be evaluated in this article, or claim that may be made by its manufacturer, is not guaranteed or endorsed by the publisher.
